# Transcription factor defects in inborn errors of immunity with atopy

**DOI:** 10.3389/falgy.2023.1237852

**Published:** 2023-09-01

**Authors:** Maryam Vaseghi-Shanjani, Pariya Yousefi, Mehul Sharma, Simran Samra, Erika Sifuentes, Stuart E. Turvey, Catherine M. Biggs

**Affiliations:** ^1^ British Columbia Children’s Hospital, Department of Pediatrics, The University of British Columbia, Vancouver, BC, Canada; ^2^ Experimental Medicine Program, Faculty of Medicine, The University of British Columbia, Vancouver, BC, Canada

**Keywords:** inborn errors of immunity, primary atopic disorders, allergy, atopy, transcription factor, monogenic allergic disease, precision medicine, primary immunodeficiency

## Abstract

Transcription factors (TFs) are critical components involved in regulating immune system development, maintenance, and function. Monogenic defects in certain TFs can therefore give rise to inborn errors of immunity (IEIs) with profound clinical implications ranging from infections, malignancy, and in some cases severe allergic inflammation. This review examines TF defects underlying IEIs with severe atopy as a defining clinical phenotype, including STAT3 loss-of-function, STAT6 gain-of-function, FOXP3 deficiency, and T-bet deficiency. These disorders offer valuable insights into the pathophysiology of allergic inflammation, expanding our understanding of both rare monogenic and common polygenic allergic diseases. Advances in genetic testing will likely uncover new IEIs associated with atopy, enriching our understanding of molecular pathways involved in allergic inflammation. Identification of monogenic disorders profoundly influences patient prognosis, treatment planning, and genetic counseling. Hence, the consideration of IEIs is essential for patients with severe, early-onset atopy. This review highlights the need for continued investigation into TF defects to enhance our understanding and management of allergic diseases.

## Introduction

Inborn errors of immunity (IEIs) are a group of disorders in which parts of the human immune system are missing or dysfunctional, predisposing to infections, autoimmunity, inflammation, and malignancy (1). It is now appreciated that certain IEIs can predominantly cause severe and early-onset allergic disease, such as asthma, food and drug allergy, atopic dermatitis, and eosinophilic gastrointestinal disease (2). IEIs associated with atopy, also referred to as primary atopic disorders, are clinically and genetically heterogeneous with more than 48 known monogenic causes identified to date (3–5). The pathogenic underpinnings vary depending on the affected gene, spanning alterations of skin epithelial barrier function, through disruptions in cellular metabolism or actin cytoskeleton function, to defects in transcription factors (TFs) or signaling molecules important for immune cell development or function (4). There is a critical need to understand the mechanisms underlying atopic immune dysregulation in order to develop targeted therapeutics that can effectively address the dysregulated pathways.

In this review, we focus on monogenic allergic diseases caused by genetic variation in TFs, as within this group lie key clinical conditions for providers to be aware of, exciting new discoveries within the field, and important lessons in immunopathogenesis of allergic disease. TFs recognize specific DNA sequences through their DNA binding domain (DBD) to control transcription (6). They act as “master regulators” controlling immune cell lineage specification and governing specific signaling pathways (7). TFs are often structurally constrained, evolutionarily conserved and depleted of common variation within their DBDs (8). The variant alleles can exert effects through loss-of-function (LOF, e.g., non-functional protein product), gain-of-function (GOF, e.g., altering sequence recognition/strength or through constitutive activation), or dominant negative [DN, e.g., interfering with the wild-type (WT) allele through disrupted dimerization or off-target binding] mechanisms—or potentially through a combination of a number of these pathogenic mechanisms (9). TF defects can disrupt the expression of genes involved in immune system development, activation, and differentiation, leading to different IEI manifestations, including atopy (4, 5, 10). In this review, we will explore major classes of TFs implicated in monogenic allergic diseases, describing their role in immune cell function and human disease. We highlight the key clinical features of IEIs associated with atopy caused by TF defects, and therapies to consider for affected individuals.

### Transcription factors orchestrating allergic immune responses

**Figure 1:**
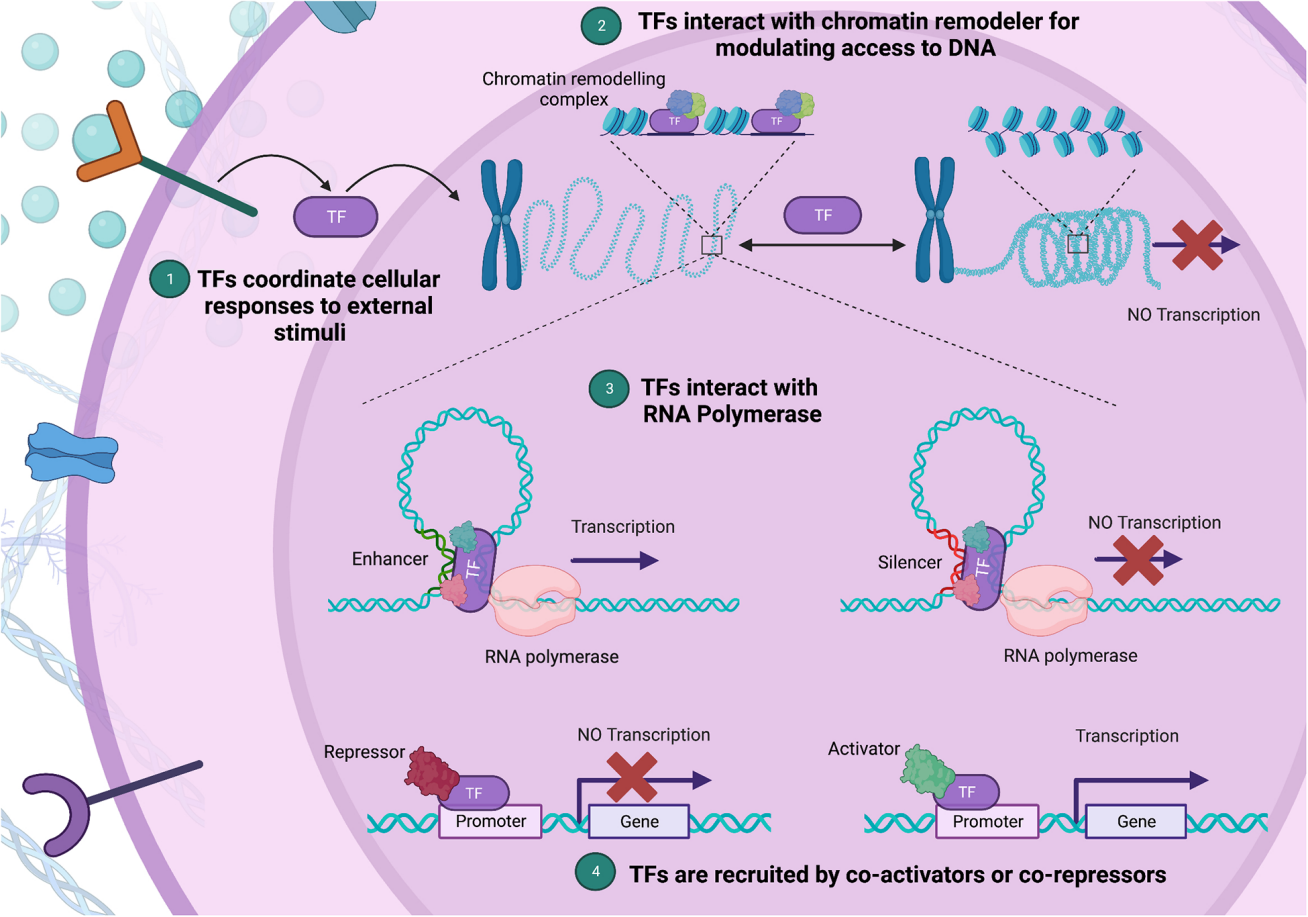
Diverse mechanisms of gene expression regulation by transcription factors (TFs). (1) TFs integrate external stimuli, coordinating cellular responses through gene expression changes. (2) Upon binding to a specific DNA sequence, a TF can act as a bridge or docking site for chromatin remodeling complexes, aiding their recruitment to nearby nucleosomes. (3) By facilitating a process called “looping”, TFs bring the promoter region, where transcription initiates, into proximity with enhancer or silencer regions, which house TF binding sites. (4) Lastly, TFs can alter gene expression by interacting with co-activators and co-repressors *via* their protein-binding domains. In this multi-pronged manner, TFs form complex regulatory networks, shaping chromatin structure and guiding the transcriptional machinery. (Created with Biorender.com).

Transcription factors (TFs) function as central regulators of cellular functions, governing gene transcription through several known mechanisms (Figure 1) (11). TFs are proteins that bind to specific DNA sequences known as TF-binding sites that are situated in the promoter or enhancer regions of genes. Transcriptional regulation is mediated through recruitment of either co-activator or co-repressor accessory proteins which either enhance or inhibit transcriptional activity, respectively (12). Additionally, TFs can interact with chromatin remodelers to modify the structure of nucleosomes, which can affect the accessibility of DNA to transcriptional regulators. These proteins can also interact with RNA polymerase and either stimulate or inhibit its activity, leading to changes in transcriptional output. Lastly, TFs can integrate signals from various signaling pathways, such as growth factors, cytokines, or hormones, and modify gene expression accordingly. The specific mechanisms by which TFs regulate gene expression is context-dependent and as such varies between genes and cell types (6, 11, 13). Given their central role in regulating gene expression, minor variations in their expression and/or function can precipitate substantial disturbances in the gene regulation network. Furthermore, TFs require precision in their expression levels due to dosage sensitivity (14). This highlights the exactness required in TF expression and function, and the significant potential for harm from pathogenic variants in TF-encoding genes (15).

Transcription factors play central roles in immune system development, maintenance, and function. These factors not only regulate cell-fate determination, but also coordinate immune cell responses following exposure to both endogenous and exogenous stimuli such as pathogens and allergens (16). In atopic disorders, a complex network of genes and their products are regulated by a host of TFs to induce allergic inflammation, which involves the recruitment and activation of various immune cells, such as mast cells, eosinophils, and T-helper 2 (Th2) lymphocytes. This ultimately leads to the production of pro-inflammatory cytokines, chemokines, and allergen-specific IgE antibodies, which collectively contribute to the hallmark clinical features of allergic disease. Key TFs involved in this process are GATA-3, STAT6, NF-κB, T-bet, AP-1, and NFAT (17). While these TFs have been shown to play crucial roles in modulating allergic immune responses, we will focus our discussion on TFs that have been confirmed to cause monogenic allergic disease.

### Transcription factors implicated in the pathophysiology of monogenic allergic diseases

Given the significant role that TFs play in modulating immune responses, it is not surprising that defects in TFs or their regulators have been linked to numerous human diseases, including approximately 25% of monogenic allergic disease (Table 1) (4, 18).

**Table 1:** Immunologic and clinical features of transcription factor defects in inborn errors of immunity associated with atopy.

Transcription factor (genetics)	Key Protein Functions	Immunological Mechanisms	Clinical Manifestations
STAT1 GOF (AD)	- Signal transducer and transcription factor (19) - Major mediator of the cellular response to interferons (IFNs) (19)	- Increased cellular responses to STAT1-dependent cytokines (IFN-α/β, IFN-γ, and IL-27) (20) - Impaired IL-17A and IL-22 production (21) - Impaired tolerogenic functions and proinflammatory skewing of DCs (22)	Eczema, CMC, infections, autoimmunity, enteropathy, malignancy, vasculopathy
STAT3 LOF (AD, DN)	- Signal transducer and transcription factor (23) - Mediates cellular responses to cytokines including IL-6, IL-10, IL-21, and IL-23 (23) - Promotes differentiation of Th17 CD4 T cells (23) - Controls development of Tfh cells and germinal center formation (23)	- Impaired IL-6 signaling (23) - Impaired Th17 differentiation (23) - Skewing towards Th2 differentiation (25) - Defective IL-10 signaling and impaired immune tolerance (24, 25) - Eosinophilia, elevated IgE	Eczema, bacterial skin abscesses, sinopulmonary infections, CMC, bone and connective tissue abnormalities, lymphoma
STAT3 GOF (AD)	- Signal transducer and transcription factor (23) - Mediates cellular responses to cytokines including IL-6, IL-10, IL-21, and IL-23 (23) - Promotes differentiation of Th17 CD4 T cells (23) - Controls development of Tfh cells and germinal center formation (23)	- Decreased Tregs and lower expression of FOXP3 and/or CD25 on Tregs (26, 27) - Elevated αβ^+^ double negative T cells (28) - Oligoclonal accumulation of effector CD8 T cells (29)	Eczema, lymphoproliferation, autoimmunity, infections, enteropathy, vasculopathy, growth impairment
STAT5B LOF (AD, DN or AR)	- Signal transducer and transcription factor - Promotes expression of *FOXP3*, *CD25*, and *IGF1* (30) - Mediates cellular responses to IL-2, IL-3, IL-5, IL-7, IL-9, IL-15, and IL-21 (30)	- Decreased Tregs, γ-*δ* T cells, and NK cells (31) - Disruption of GM-CSF signaling (32) - Elevated IgE level	Eczema, growth impairment, autoimmunity, enteropathy, infections, pulmonary disease
STAT5B GOF (germline AD or somatic)	- Signal transducer and transcription factor - Promotes expression of *FOXP3*, *CD25*, and *IGF1* (30) - Mediates cellular responses to IL-2, IL-3, IL-5, IL-7, IL-9, IL-15, and IL-21 (30)	- Increased production of Th2 cytokines by CD4 T cells (33) - Increased Tregs (33, 34) - Eosinophilia, elevated IgE	Atopic dermatitis, allergy, asthma, urticaria, hair loss, enteropathy
STAT6 GOF (AD)	- Signal transducer and transcription factor - Mediates cellular responses to IL-4 and IL-13 (35) - Promotes differentiation of Th2 CD4 T cells	- Th2 skewing of CD4 T cells (36) - Eosinophilia, elevated IgE	Atopic dermatitis, allergy, EGID, asthma, anaphylaxis, infections, growth impairment, vasculopathy
FOXP3 LOF (XLR)	- Transcriptional regulator of Treg development and inhibitory function (37) - Inhibits cytokine production and T-cell effector function (38) - Mediates transcriptional repression of IL-2 (39)	- Impaired Treg function - Disrupted regulation of mTOR and glycolysis - Skewing of Tregs to “Th2-like” Teff cells - Eosinophilia, elevated IgE	Atopic dermatitis, asthma, EGID, allergy, infections, autoimmunity, and enteropathy
T-bet LOF (AR)	- Regulates the differentiation of Th1 cells through various mechanisms inducing IFN-*γ* expression (40) and repressing the development of other subsets of T helper cells (41)	- Impaired IFN-γ production by innate and innate-like adaptive lymphocytes (42) - Excessive Th2 cytokine production by CD4 T cells (42) - Eosinophilia	Upper airway hyperresponsiveness, mycobacterial disease
T-box 1 LOF* (AD, HI)	- Regulates thymic epithelium development (43)	- Severe naïve T cell lymphopenia (44) - Oligoclonal T cell expansion - Eosinophilia, elevated IgE	Eczematous dermatitis, lymphadenopathy, enteropathy, infections, hypoparathyroidism, congenital heart disease
ZNF341 LOF (AR)	- Transcription factor controlling STAT3 expression (45)	- Increased Th2 cells (46) - Decreased Tfh, Th17 and NK cells (46) - Eosinophilia, elevated IgE	Eczema, skin and respiratory tract infections, CMC, skeletal and connective tissue abnormalities

AD, autosomal dominant; AR, autosomal recessive; CMC, chronic mucocutaneous candidiasis; DN, dominant negative; EGID, eosinophilic gastrointestinal diseases; GM-CSF, granulocyte-macrophage colony-stimulating factor; GOF, gain-of-function; HI, haploinsufficiency; IFN, interferon; IGF, insulin-like growth factor; LOF, loss-of-function; NK cell, natural killer cell; Th, T helper cell; Tfh, T follicular helper cell, XLR, X-linked recessive.

* Refers to phenotype of atypical complete DiGeorge.

 In some cases, the affected TFs are important for regulating the balance between Th1 and Th2 responses, commonly referred to as the “Th1/Th2 paradigm”. An imbalance or skewing towards either Th1 or Th2 disrupts immune homeostasis and can give rise to immune-related disorders. As such, the basis of allergy can be partly understood through the lens of this delicate equilibrium, as a skewing towards Th2 immunity and allergic inflammation (47). However, it is essential to recognize that the pathogenesis of monogenic allergic diseases is not solely restricted to dysregulation of Th1/Th2 balance. As we delve further into the molecular mechanisms underlying IEIs associated with atopy, it becomes increasingly clear that genetic variants in several components of the immune system, beyond those directly related to Th1 and Th2 cells, can cause these disorders. Consequently, a comprehensive understanding of monogenic allergic diseases necessitates the consideration of multiple molecular pathways and immune system components as contributors to disease etiology.

#### STAT family of transcription factors

Monogenic defects impacting the Janus kinase-signal transducer and activator of transcription (JAK-STAT) signaling pathway comprise a key group of IEIs associated with atopy (3, 10, 48). The JAK-STAT family consists of 7 TFs (STAT1, STAT2, STAT3, STAT4, STAT5A/B, and STAT6) and 4 receptor associated kinases (JAK1, JAK2, JAK3, TYK2) that regulate various cellular functions such as immunity, growth, differentiation, and survival (49, 50). Despite each of these TFs possessing distinct functions, they exhibit sequence similarities and analogous activation mechanisms. The JAK-STAT signaling cascade is initiated upon ligand binding to an extracellular cytokine or growth factor receptor, leading to activation of the JAK kinase, phosphorylation of the receptor, and recruitment and activation of STAT proteins. The STAT proteins then dimerize and migrate to the cell nucleus, where they bind to specific DNA sequences (response elements) in the promoter regions of target genes and regulate their transcription. The combinatorial and asymmetric activation of STAT TFs in response to a cytokine stimulus enables the JAK-STAT pathway to transmit over 50 unique cytokine signals and transcriptional outputs (51–55). When one STAT TF's activity is significantly altered, the transcriptome induced by the cytokines upstream of its action will change. This leads to abnormal immune responses that frequently skew towards Th2, resulting in the atopic phenotype seen in IEIs caused by genetic variants in STATs including *STAT1*, *STAT3*, *STAT5b* and *STAT6* (Table 1) (33, 36, 56, 57). The phenotypic variability seen between and within STAT TF defects stems not just from the variant's functional impact—whether LOF, GOF, or dominant negative (DN)—but also from which TFs' domain was genetically altered. Thus, understanding how these variants directly affect TF functionality, and consequently the JAK-STAT pathway, is crucial to comprehending the broad spectrum of clinical manifestations these diseases present.

**Figure 2:**
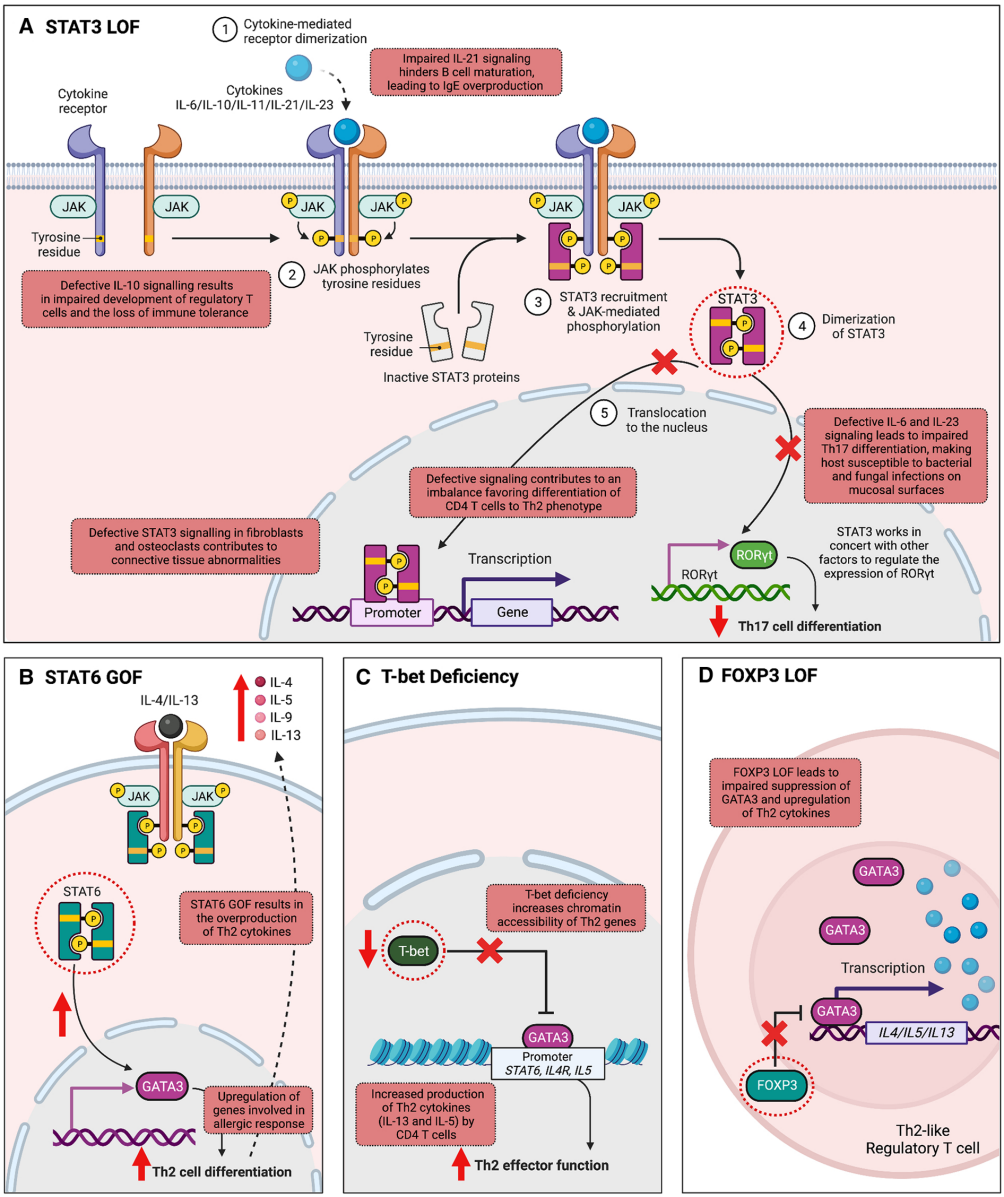
Mechanisms by which variants in transcription factor-encoding genes cause inborn errors of immunity associated with atopy. (**A**) Signal transducer and activator of transcription 3 (STAT3) is integral to the transduction of multiple cytokine signals, including IL-6, IL-10, IL-11, IL-21, IL-22, and IL-23. Autosomal dominant loss-of-function (LOF) in STAT3 leads to abnormalities in several cytokine signaling pathways. Impaired IL-6 and IL-23 signaling hinders Th17 cell differentiation, increasing host susceptibility to mucosal bacterial and fungal infections. Additionally, IL-21 function is compromised, affecting B cell maturation and isotype switching, thereby leading to elevated IgE levels. Impaired IL-11 signaling is associated with connective tissue abnormalities. T helper 2 (Th2) skewing is observed in CD4+ T cells, and impaired IL-10 signaling is associated with impaired development of regulatory T cells (Tregs). (**B**) STAT6 plays a fundamental role in allergic inflammation, mediating the effects of cytokines essential for Th2 cell differentiation, B cell proliferation, and class switching to IgE. In T cells, STAT6 activation upregulates *GATA Binding Protein 3* (*GATA3*) expression, amplifying cytokines IL-4, IL-5, and IL-13, which stimulate allergic responses, explaining the allergic phenotype observed in STAT6 gain-of-function (GOF). (**C**) T-bet, the master regulator of Th1 differentiation, suppresses Th2 cell lineage commitment by inhibiting GATA-3 function. T-bet deficiency disrupts this equilibrium, leading to increased chromatin accessibility of Th2 genes by GATA-3, resulting in the excessive production of Th2 cytokines like IL-5 and IL-13. (**D**) Forkhead box P3 (FOXP3) is an important TF for the development, stability, and suppressive function of Tregs. FOXP3 LOF leads to a failure in repressing the Th2 transcriptional program, generating “Th2-like” Tregs. These “Th2-like” Tregs exhibit increased intra-chromosomal interactions in the Th2 locus, leading to type 2 cytokine production. (Created with Biorender.com).

One of the most well-studied IEIs associated with the JAK-STAT pathway is autosomal dominant hyper-IgE syndrome (AD-HIES)/STAT3 deficiency, characterized by eczema, eosinophilia, elevated IgE levels, mucocutaneous candidiasis, and connective tissue abnormalities (58–60). AD-HIES is caused by DN variants in STAT3, typically missense or in-frame insertion/deletion variants impacting the highly conserved DNA-binding or Src Homology 2 (SH2) domain of the protein amongst other conserved regions (59, 60). Such changes do not alter the expression levels of STAT3 protein, but do hinder STAT3's ability to regulate gene transcription downstream of several key cytokines, notably IL-6, IL-10, IL-11 and IL-21 (Figure 2A) (61, 62). Defective IL-6 signaling leads to blunted inflammatory responses and impaired Th17 cell differentiation, with clinical features of recurrent staphylococcal and fungal infections. CD4+ T cells show Th2 skewing and production of IL-4, IL-5, IL-13, contributing to the atopic phenotype seen in these patients of eczema and eosinophilia. IL-21 is important for B cell maturation and class switching, while both IL-21 and IL-10 play a role in suppressing IgE production; impaired IL-10 and IL-21 responses thus lead to humoral defects and elevated IgE (61, 63). Lastly, impaired IL-11 signaling likely contributes to the delayed primary tooth exfoliation and distinctive facial features associated with AD-HIES (61, 64). Pathogenic germline variants in other molecules that regulate the expression and function of STAT3 have now been shown to be associated with severe atopic disease, signifying the central role for STAT3 in these disorders. These include Erbin (encoded by *ERBB2IP*), ZNF341 (encoded by *ZNF341*), and transforming growth factor beta (TGF-β) receptors (*TGFBR1* and *TGFBR2*) (65, 66). Management of AD-HIES commonly requires antimicrobial prophylaxis and immunoglobulin replacement to prevent infectious exacerbations. Monoclonal antibodies targeting allergic immune effectors, including omalizumab (IgE), dupilumab (IL-4 and IL-13), reslizumab, benralizumab, and mepolizumab (IL-5), have shown efficacy in treating the eczema and other allergic manifestations (67). Hematopoietic stem cell transplantation (HSCT) can restore immune function and improve rates of infection and dermatological symptoms of these patients, especially when carried out at an early age (68–70).

While LOF in certain STATs leads to allergic immune dysregulation as observed in AD-HIES, enhanced STAT TF activity can also cause severe atopy, with a notable example being the recently described IEI autosomal dominant STAT6 GOF (Figure 2B). The clinical phenotype associated with STAT6 GOF includes treatment-resistant atopic dermatitis, hypereosinophilia, eosinophilic gastrointestinal disease, asthma, elevated serum IgE, IgE-mediated food allergies, and anaphylaxis (36, 71–73). Infections, growth impairment and vascular malformations of the brain have also been reported (36). The condition is caused by heterozygous missense variants located in several protein domains including the DNA-binding, linker-, and SH2 domain of STAT6. Despite their diverse locations, most of these variants are positioned near the TF-DNA interface. By increasing the electro-positivity at this interface, they are predicted to enhance STAT6 binding to DNA, thus conferring the GOF mechanism of pathogenicity. STAT6 is fundamentally involved in allergic inflammation processes. Its pivotal role includes mediating the effects of IL-4 and IL-13, cytokines essential for Th2 cell differentiation, B cell proliferation, survival, and class switching to IgE. In T cells, STAT6 activation upregulates *GATA3* expression, a critical regulator of Th2 differentiation, which subsequently amplifies the expression of cytokines IL-4, IL-5, and IL-13. These cytokines stimulate allergic responses by activating mast cells and eosinophils. Thus, Th2 skewing and elevated levels of IL-4/IL-5/IL-13 produced by these cells could be the driving factor behind the allergic phenotype observed in STAT6 GOF. Precision treatment with the anti–IL-4Rα antibody, dupilumab, as well as JAK inhibitors have proven highly effective in improving both clinical manifestations and immune biomarkers of patients with STAT6 GOF (36, 73).

#### T-box transcription factor 21 (T-bet)

Transcription factors such as T-box transcription factor 21 (TBX21, also called T-bet) and GATA3 play pivotal roles in directing Th1 and Th2 differentiation, respectively. Aberrations in their signaling pathways or variants in genes encoding these TFs may contribute to the development of monogenic allergic diseases by disrupting the delicate equilibrium between Th1 and Th2 responses. The Th1/Th2 counter-regulatory theory is supported by a recent study describing an individual with an autosomal recessive LOF variant in *TBX21* (42), encoding T-bet, the master regulator of Th1 differentiation (74). T-bet deficiency results in the loss of IFN-γ production, and in turn is linked to the excessive production of IL-5 and IL-13 by Th2 cells, leading to the development of upper airway allergic inflammation and eosinophilia (Figure 2C) (42). The Th2 skewing and the resulting atopic phenotype observed in this patient can be attributed to the known mechanism of action of T-bet. T-bet acts as a repressor of Th2 cell lineage commitment by preventing GATA3 from binding to its target DNA and suppressing GATA3 expression (41). Through these mechanisms, T-bet effectively suppresses the production of Th2 cytokines, including IL-4, IL-5, IL-9, and IL-13, which are crucial for Th2 cell function and the development of allergic responses. Therefore, it is expected that the loss of T-bet leads to an overactive Th2 response and the subsequent development of atopic conditions such as upper airway allergic inflammation and eosinophilia (42).

#### Forkhead Box P3

FOXP3 (Forkhead Box P3) is a TF that plays a critical role in maintaining immune homeostasis. It primarily controls the differentiation and function of regulatory T cells (Tregs), a subset of T cells that prevent harmful immune responses against self and environmental antigens. Pathogenic variants in *FOXP3* cause IPEX syndrome (immune dysregulation, polyendocrinopathy, enteropathy, X-linked) (75), an IEI characterized by autoimmunity, lymphoproliferation and severe atopy. Affected individuals often present with severe atopic dermatitis, high IgE, and eosinophilia (10). Currently over 70 variants in *FOXP3* associated with IPEX have been reported (76), with the majority impacting the forkhead (FKH) DNA-binding domain of FOXP3 at the C-terminal end of the protein. Some variants are located in other FOXP3 regions, including the N-terminal proline-rich (PRR) or leucine-zipper (LZ) domains, whereas others are located upstream of the gene or in the polyadenylation site, thus influencing mRNA expression and/or stability. While lack of FOXP3 expression has been linked to severe phenotypes, disease severity does not always correlate with protein expression. Many of the affected individuals have missense variants that lead to normal or reduced expression of the variant protein, disrupting the regulatory activity of FOXP3 by changing its DNA binding sites, interactions with other molecules, or its ability to form dimers (77).

FOXP3-deficient Tregs still develop in the thymus however they lose their regulatory function and acquire effector T cell (Teff) attributes (78, 79). The balance of T cell fate between Treg and Teff phenotype is in part related to metabolic programming which FOXP3 mediates. Teff cells are highly metabolically active, undergoing glycolysis and oxidative phosphorylation, whereas Treg metabolism favours fatty acid oxidative and keeps glycolysis under strict control. FOXP3 deficiency impairs its ability to regulate the metabolic kinase mammalian target of rapamycin (mTOR), leading to augmented glycolysis and degeneration of Tregs into Teff cells without suppressive function (79).

Several studies provide further insight into how alterations or loss of FOXP3 drives atopy (Figure 2D). One study recapitulated in a mouse model a human IPEX syndrome-causing variant (M370I) impacting the FKH domain of FOXP3 (80). Compared to wild-type Tregs, M370I Tregs were much less efficient at inhibiting GATA3 expression during T cell activation, thus skewing extrinsic CD4+ cells towards Th2 differentiation. Moreover, M370I Tregs were unable to repress Th2 transcriptional programs intrinsically, leading to generation of “Th2-like” Tregs with an effector Th2 phenotype and type 2 cytokine production. This is in keeping with previous work demonstrating FOXP3's role in preventing degeneration of Tregs into effector T cells. A separate study similarly demonstrated that downregulation of FOXP3 in human Tregs is associated with strong and selective upregulation of Th2 signature genes, such as GATA-3, IL-4, IL-5 and IL-13, supporting Th2 as the default differentiation pathway of FOXP3-negative Tregs (81).

Management of IPEX syndrome involves avoidance of immune triggers such as infections, immunosuppressive therapy to control aberrant immune responses, and HSCT. Immunomodulatory therapy with agents such as rapamycin are crucial in managing the overactive immune response and reducing inflammation and autoimmunity associated with the syndrome (76, 82). Rapamycin provides benefit in IPEX syndrome by partially restoring Treg function independent of *FOXP3* expression or Treg frequency (82). A selective mTOR inhibitor, rapamycin induces a metabolic switch in FOXP3-deficient Tregs that suppresses the Teff-like reprogramming and restores their suppressive capacity (79, 82). HSCT represents a potentially curative treatment option for IPEX syndrome by replacing the dysfunctional immune system with that of a healthy donor (83).

## Discussion

Transcription factors play a central role in immune cell responses and function. They integrate signals to regulate gene transcription in response to a stimulus, thus shaping the differentiation and phenotype of key immune cells. When the function of a TF involved in immune responses is altered, such as in the setting of a monogenic defect, profound clinical implications may arise. In this review, we highlighted key IEIs associated with atopy caused by TF defects, including STAT3 LOF, STAT6 GOF, FOXP3 deficiency, and T-bet deficiency. Studying these conditions has provided remarkable insights into the pathophysiology of allergic inflammation, with implications not only for the rare conditions themselves but for common allergic diseases as well. Given their indispensable role in immunity, we anticipate the identification of more forms of human IEIs caused by TF defects. Focusing on IEIs associated with atopy, we can consider other key TFs in whom a monogenic immune disorder has not yet been described, but that would be expected to have an atopic phenotype. Examples of these would include GATA3, a TF that regulates Th2 differentiation, as well as STAT4 (LOF) and BCL-6 that are also important for inhibiting or promoting a Th2 phenotype. As access to genetic testing expands, the global community is very likely to find new causes of IEIs with atopy, which may lead to some anticipated and unanticipated lessons on the factors governing allergic inflammation. Due to the important implications that identifying a monogenic disorder has on prognosis, treatment planning and genetic counselling, it is imperative that clinicians continue to consider IEIs on the differential of individuals who present with severe, early-onset atopy.

## References

[1] Turvey SE Bonilla FA Junker AK. Primary immunodeficiency diseases: a practical guide for clinicians. Postgrad Med J. (2009) 85(1010):660–6. 10.1136/pgmj.2009.080630 20075404

[2] Castagnoli R Lougaris V Giardino G Volpi S Leonardi L La Torre F Inborn errors of immunity with atopic phenotypes: a practical guide for allergists. World Allergy Organ J. (2021) 14(2):100513. 10.1016/j.waojou.2021.100513 33717395 PMC7907539

[3] Vaseghi-Shanjani M Smith KL Sara RJ Modi BP Branch A Sharma M Inborn errors of immunity manifesting as atopic disorders. J Allergy Clin Immunol. (2021) 148(5):1130–9. 10.1016/j.jaci.2021.08.008 34428518

[4] Vaseghi-Shanjani M Snow AL Margolis DJ Latrous M Milner JD Turvey SE Atopy as immune dysregulation: offender genes and targets. J Allergy Clin Immunol Pract. (2022) 10(7):1737–56. 10.1016/j.jaip.2022.04.001 35680527

[5] Nelson RW Geha RS McDonald DR. Inborn errors of the immune system associated with atopy. Front Immunol. (2022) 13:860821. 10.3389/fimmu.2022.860821 35572516 PMC9094424

[6] Lambert SA Jolma A Campitelli LF Das PK Yin Y Albu M The human transcription factors. Cell. (2018) 175(2):598–9. 10.1016/j.cell.2018.09.045 30290144

[7] Singh H Khan AA Dinner AR. Gene regulatory networks in the immune system. Trends Immunol. (2014) 35(5):211–8. 10.1016/j.it.2014.03.006 24768519

[8] Barrera LA Vedenko A Kurland JV Rogers JM Gisselbrecht SS Rossin EJ Survey of variation in human transcription factors reveals prevalent DNA binding changes. Science. (2016) 351(6280):1450–4. 10.1126/science.aad2257 27013732 PMC4825693

[9] Fornes O Jia A Kuehn HS Min Q Pannicke U Schleussner N A multimorphic mutation in IRF4 causes human autosomal dominant combined immunodeficiency. Sci Immunol. (2023) 8(79):eade7953. 10.1126/sciimmunol.ade7953 36662884 PMC10825898

[10] Lyons JJ Milner JD. Primary atopic disorders. J Exp Med. (2018) 215(4):1009–22. 10.1084/jem.20172306 29549114 PMC5881472

[11] Stampfel G Kazmar T Frank O Wienerroither S Reiter F Stark A. Transcriptional regulators form diverse groups with context-dependent regulatory functions. Nature. (2015) 528(7580):147–51. 10.1038/nature15545 26550828

[12] Reiter F Wienerroither S Stark A. Combinatorial function of transcription factors and cofactors. Curr Opin Genet Dev. (2017) 43:73–81. 10.1016/j.gde.2016.12.007 28110180

[13] Frietze S Farnham PJ. Transcription factor effector domains. Subcell Biochem. (2011) 52:261–77. 10.1007/978-90-481-9069-0_12 21557087 PMC4151296

[14] Van Der Lee R Correard S Wasserman WW. Deregulated regulators: disease-causing cis variants in transcription factor genes. Trends Genet. (2020) 36(7):523–39. 10.1016/j.tig.2020.04.006 32451166

[15] Latchman DS. Transcription-factor mutations and disease. N Engl J Med. (1996) 334(1):28–33. 10.1056/NEJM199601043340108 7494569

[16] Smale ST. Transcriptional regulation in the immune system: a status report. Trends Immunol. (2014) 35(5):190–4. 10.1016/j.it.2014.03.003 24703179 PMC4041609

[17] Escoubet-Lozach L Glass CK Wasserman SI. The role of transcription factors in allergic inflammation. J Allergy Clin Immunol. (2002) 110(4):553–64. 10.1067/mai.2002.128076 12373260

[18] Tangye SG Al-Herz W Bousfiha A Cunningham-Rundles C Franco JL Holland SM Human inborn errors of immunity: 2022 update on the classification from the international union of immunological societies expert committee. J Clin Immunol. (2022) 42(7):1473–507. 10.1007/s10875-022-01289-3 35748970 PMC9244088

[19] Tolomeo M Cavalli A Cascio A. STAT1 and its crucial role in the control of viral infections. Int J Mol Sci. (2022) 23(8):4095. 10.3390/ijms23084095 35456913 PMC9028532

[20] Liu L Okada S Kong X-F Kreins AY Cypowyj S Abhyankar A Gain-of-function human STAT1 mutations impair IL-17 immunity and underlie chronic mucocutaneous candidiasis. J Exp Med. (2011) 208(8):1635–48. 10.1084/jem.20110958 21727188 PMC3149226

[21] Yamazaki Y Yamada M Kawai T Morio T Onodera M Ueki M Two novel gain-of-function mutations of STAT1 responsible for chronic mucocutaneous candidiasis disease: impaired production of IL-17A and IL-22, and the presence of anti-IL-17F autoantibody. J Immunol. (2014) 193(10):4880–7. 10.4049/jimmunol.1401467 25288569

[22] Parackova Z Zentsova I Vrabcova P Sediva A Bloomfield M. Aberrant tolerogenic functions and proinflammatory skew of dendritic cells in STAT1 gain-of-function patients may contribute to autoimmunity and fungal susceptibility. Clin Immunol. (2023) 246:109174. 10.1016/j.clim.2022.109174 36372319

[23] Hillmer EJ Zhang H Li HS Watowich SS. STAT3 signaling in immunity. Cytokine Growth Factor Rev. (2016) 31:1–15. 10.1016/j.cytogfr.2016.05.001 27185365 PMC5050093

[24] Saito M Nagasawa M Takada H Hara T Tsuchiya S Agematsu K Defective IL-10 signaling in hyper-IgE syndrome results in impaired generation of tolerogenic dendritic cells and induced regulatory T cells. J Exp Med. (2011) 208(2):235–49. 10.1084/jem.20100799 21300911 PMC3039860

[25] Khourieh J Rao G Habib T Avery DT Lefèvre-Utile A Chandesris MO A deep intronic splice mutation of STAT3 underlies hyper IgE syndrome by negative dominance. Proc Natl Acad Sci U S A. (2019) 116(33):16463–72. 10.1073/pnas.1901409116 31346092 PMC6697804

[26] Milner JD Vogel TP Forbes L Ma CA Stray-Pedersen A Niemela JE Early-onset lymphoproliferation and autoimmunity caused by germline STAT3 gain-of-function mutations. Blood. (2015) 125(4):591–9. 10.1182/blood-2014-09-602763 25359994 PMC4304103

[27] Vogel TP Leiding JW Cooper MA Forbes Satter LR. STAT3 gain-of-function syndrome. Front Pediatr. (2022) 10:770077. 10.3389/fped.2022.770077 36843887 PMC9948021

[28] Faletti L Ehl S Heeg M. Germline STAT3 gain-of-function mutations in primary immunodeficiency: impact on the cellular and clinical phenotype. Biomed J. (2021) 44(4):412–21. 10.1016/j.bj.2021.03.003 34366294 PMC8514798

[29] Masle-Farquhar E Jackson KJL Peters TJ Al-Eryani G Singh M Payne KJ STAT3 gain-of-function mutations connect leukemia with autoimmune disease by pathological NKG2D(hi) CD8(+) T cell dysregulation and accumulation. Immunity. (2022) 55(12):2386–404 e8. 10.1016/j.immuni.2022.11.001 36446385

[30] Smith MR Satter LRF Vargas-Hernandez A. STAT5b: a master regulator of key biological pathways. Front Immunol. (2022) 13:1025373. 10.3389/fimmu.2022.1025373 36755813 PMC9899847

[31] Kanai T Jenks J Nadeau KC. The STAT5b pathway defect and autoimmunity. Front Immunol. (2012) 3:234. 10.3389/fimmu.2012.00234 22912632 PMC3418548

[32] KroneKAFoleyCLFishmanMPVargasSOForbesLRVeceTJ Signal transducer and activator of transcription 5B deficiency-associated lung disease. Am J Respir Crit Care Med. (2022) 205(10):1245–50. 10.1164/rccm.202111-2527LE35238729

[33] Ma CA Xi L Cauff B Dezure A Freeman AF Hambleton S Somatic STAT5b gain-of-function mutations in early onset nonclonal eosinophilia, urticaria, dermatitis, and diarrhea. Blood. (2017) 129(5):650–3. 10.1182/blood-2016-09-737817 27956386 PMC5290989

[34] Kasap N Aslan K Karakurt LT Bozkurt H Canatan H Cavkaytar O A novel gain-of-function mutation in STAT5B is associated with treatment-resistant severe atopic dermatitis. Clin Exp Allergy. (2022) 52(7):907–10. 10.1111/cea.14148 35426955

[35] Goenka S Kaplan MH. Transcriptional regulation by STAT6. Immunol Res. (2011) 50(1):87–96. 10.1007/s12026-011-8205-2 21442426 PMC3107597

[36] Sharma M Leung D Momenilandi M Jones LCW Pacillo L James AE Human germline heterozygous gain-of-function STAT6 variants cause severe allergic disease. J Exp Med. (2023) 220(5):e20221755. 10.1084/jem.20221755 36884218 PMC10037107

[37] Bandukwala HS Wu Y Feuerer M Chen Y Barboza B Ghosh S Structure of a domain-swapped FOXP3 dimer on DNA and its function in regulatory T cells. Immunity. (2011) 34(4):479–91. 10.1016/j.immuni.2011.02.017 21458306 PMC3085397

[38] Bettelli E Dastrange M Oukka M. Foxp3 interacts with nuclear factor of activated T cells and NF-κB to repress cytokine gene expression and effector functions of T helper cells. Proc Natl Acad Sci USA. (2005) 102(14):5138–43. 10.1073/pnas.0501675102 15790681 PMC555574

[39] Li B Samanta A Song X Iacono KT Bembas K Tao R FOXP3 interactions with histone acetyltransferase and class II histone deacetylases are required for repression. Proc Natl Acad Sci USA. (2007) 104(11):4571–6. 10.1073/pnas.0700298104 17360565 PMC1838642

[40] Miller SA Weinmann AS. Molecular mechanisms by which T-bet regulates T-helper cell commitment. Immunol Rev. (2010) 238(1):233–46. 10.1111/j.1600-065X.2010.00952.x 20969596 PMC2988494

[41] Zhu J Jankovic D Oler AJ Wei G Sharma S Hu G The transcription factor T-bet is induced by multiple pathways and prevents an endogenous Th2 cell program during Th1 cell responses. Immunity. (2012) 37(4):660–73. 10.1016/j.immuni.2012.09.007 23041064 PMC3717271

[42] Yang R Weisshaar M Mele F Benhsaien I Dorgham K Han J High Th2 cytokine levels and upper airway inflammation in human inherited T-bet deficiency. J Exp Med. (2021) 218(8):e20202726. 10.1084/jem.20202726 34160550 PMC8225679

[43] Gao S Li X Amendt BA. Understanding the role of Tbx1 as a candidate gene for 22q11.2 deletion syndrome. Curr Allergy Asthma Rep. (2013) 13(6):613–21. 10.1007/s11882-013-0384-6 23996541 PMC3840116

[44] Markert ML Alexieff MJ Li J Sarzotti M Ozaki DA Devlin BH Complete DiGeorge syndrome: development of rash, lymphadenopathy, and oligoclonal T cells in 5 cases. J Allergy Clin Immunol. (2004) 113(4):734–41. 10.1016/j.jaci.2004.01.766 15100681

[45] Frey-Jakobs S Hartberger JM Fliegauf M Bossen C Wehmeyer ML Neubauer JC ZNF341 controls STAT3 expression and thereby immunocompetence. Sci Immunol. (2018) 3(24):eaat4941. 10.1126/sciimmunol.aat4941 29907690 PMC6173313

[46] Beziat V Fieschi C Momenilandi M Migaud M Belaid B Djidjik R Inherited human ZNF341 deficiency. Curr Opin Immunol. (2023) 82:102326. 10.1016/j.coi.2023.102326 37080116 PMC10620851

[47] Mosmann TR Coffman RL. TH1 and TH2 cells: different patterns of lymphokine secretion lead to different functional properties. Annu Rev Immunol. (1989) 7:145–73. 10.1146/annurev.iy.07.040189.001045 2523712

[48] Milner JD. Primary atopic disorders. Annu Rev Immunol. (2020) 38:785–808. 10.1146/annurev-immunol-042718-041553 32126183

[49] O’Shea JJ Plenge R. JAK and STAT signaling molecules in immunoregulation and immune-mediated disease. Immunity. (2012) 36(4):542–50. 10.1016/j.immuni.2012.03.014 22520847 PMC3499974

[50] Luo Y Alexander M Gadina M O’Shea JJ Meylan F Schwartz DM. JAK-STAT signaling in human disease: from genetic syndromes to clinical inhibition. J Allergy Clin Immunol. (2021) 148(4):911–25. 10.1016/j.jaci.2021.08.004 34625141 PMC8514054

[51] Ihle JN. Cytokine receptor signalling. Nature. (1995) 377(6550):591–4. 10.1038/377591a0 7566171

[52] Mogensen TH. IRF and STAT transcription factors - from basic biology to roles in infection, protective immunity, and primary immunodeficiencies. Front Immunol. (2018) 9:3047. 10.3389/fimmu.2018.03047 30671054 PMC6331453

[53] Briscoe J Guschin D Rogers NC Watling D Muller M Horn F JAKs, STATs and signal transduction in response to the interferons and other cytokines. Philos Trans R Soc Lond B Biol Sci. (1996) 351(1336):167–71. 10.1098/rstb.1996.0013 8650263

[54] Villarino AV Kanno Y Ferdinand JR O’Shea JJ. Mechanisms of Jak/STAT signaling in immunity and disease. J Immunol. (2015) 194(1):21–7. 10.4049/jimmunol.1401867 25527793 PMC4524500

[55] Hirahara K Onodera A Villarino AV Bonelli M Sciumè G Laurence A Asymmetric action of STAT transcription factors drives transcriptional outputs and cytokine specificity. Immunity. (2015) 42(5):877–89. 10.1016/j.immuni.2015.04.014 25992861 PMC11037422

[56] Holland SM DeLeo FR Elloumi HZ Hsu AP Uzel G Brodsky N STAT3 mutations in the hyper-IgE syndrome. N Engl J Med. (2007) 357(16):1608–19. 10.1056/NEJMoa073687 17881745

[57] Uzel G Sampaio EP Lawrence MG Hsu AP Hackett M Dorsey MJ Dominant gain-of-function STAT1 mutations in FOXP3 wild-type immune dysregulation-polyendocrinopathy-enteropathy-X-linked-like syndrome. J Allergy Clin Immunol. (2013) 131(6):1611–23. 10.1016/j.jaci.2012.11.054 23534974 PMC3672257

[58] Davis SD Schaller J Wedgwood RJ. Job’s syndrome. Recurrent, “cold”, staphylococcal abscesses. Lancet. (1966) 1(7445):1013–5. 10.1016/s0140-6736(66)90119-x 4161105

[59] Grimbacher B Holland SM Gallin JI Greenberg F Hill SC Malech HL Hyper-IgE syndrome with recurrent infections–an autosomal dominant multisystem disorder. N Engl J Med. (1999) 340(9):692–702. 10.1056/NEJM199903043400904 10053178

[60] Asano T Khourieh J Zhang P Rapaport F Spaan AN Li J Human STAT3 variants underlie autosomal dominant hyper-IgE syndrome by negative dominance. J Exp Med. (2021) 218(8):e20202592. 10.1084/jem.20202592 34137790 PMC8217968

[61] Al-Shaikhly T Ochs HD. Hyper IgE syndromes: clinical and molecular characteristics. Immunol Cell Biol. (2019) 97(4):368–79. 10.1111/imcb.12209 30264496

[62] Minegishi Y Saito M Tsuchiya S Tsuge I Takada H Hara T Dominant-negative mutations in the DNA-binding domain of STAT3 cause hyper-IgE syndrome. Nature. (2007) 448(7157):1058–62. 10.1038/nature06096 17676033

[63] Mogensen TH. STAT3 and the hyper-IgE syndrome: clinical presentation, genetic origin, pathogenesis, novel findings and remaining uncertainties. Jak-Stat. (2013) 2(2):e23435. 10.4161/jkst.23435 24058807 PMC3710320

[64] Nieminen P Morgan NV Fenwick AL Parmanen S Veistinen L Mikkola ML Inactivation of IL11 signaling causes craniosynostosis, delayed tooth eruption, and supernumerary teeth. Am J Hum Genet. (2011) 89(1):67–81. 10.1016/j.ajhg.2011.05.024 21741611 PMC3135804

[65] Lyons JJ Liu Y Ma CA Yu X O'Connell MP Lawrence MG ERBIN deficiency links STAT3 and TGF-β pathway defects with atopy in humans. J Exp Med. (2017) 214(3):669–80. 10.1084/jem.20161435 28126831 PMC5339676

[66] Béziat V Li J Lin JX Ma CS Li P Bousfiha A A recessive form of hyper-IgE syndrome by disruption of ZNF341-dependent STAT3 transcription and activity. Sci Immunol. (2018) 3(24):eaat4956. 10.1126/sciimmunol.aat4956 29907691 PMC6141026

[67] James AE West L Schloss K Nataraj P Urban A Hirsch A Treatment of STAT3-deficient hyper-immunoglobulin E syndrome with monoclonal antibodies targeting allergic inflammation. J Allergy Clin Immunol Pract. (2022) 10(5):1367–70 e1. 10.1016/j.jaip.2022.01.011 35085810

[68] Oikonomopoulou C Goussetis E. Autosomal dominant hyper-IgE syndrome: when hematopoietic stem cell transplantation should be considered? Pediatr Transplant. (2020) 24(5):e13699. 10.1111/petr.13699 32497403

[69] Harrison SC Tsilifis C Slatter MA Nademi Z Worth A Veys P Hematopoietic stem cell transplantation resolves the immune deficit associated with STAT3-dominant-negative hyper-IgE syndrome. J Clin Immunol. (2021) 41(5):934–43. 10.1007/s10875-021-00971-2 33523338 PMC8249289

[70] Ponsford MJ Clark J Mock J Abinun M Carne E El-Shanawany T Hematopoietic stem cell transplantation and vasculopathy associated with STAT3-dominant-negative hyper-IgE syndrome. Front Pediatr. (2020) 8:575. 10.3389/fped.2020.00575 33014947 PMC7511721

[71] Takeuchi I Yanagi K Takada S Uchiyama T Igarashi A Motomura K STAT6 gain-of-function variant exacerbates multiple allergic symptoms. J Allergy Clin Immunol. (2023) 151(5):1402–9.e6. 10.1016/j.jaci.2022.12.802 36538978

[72] Suratannon N Ittiwut C Dik WA Ittiwut R Meesilpavikkai K Israsena N A germline STAT6 gain-of-function variant is associated with early-onset allergies. J Allergy Clin Immunol. (2023) 151(2):565–71.e9. 10.1016/j.jaci.2022.09.028 36216080

[73] Baris S Benamar M Chen Q Catak MC Martínez-Blanco M Wang M Severe allergic dysregulation due to a gain of function mutation in the transcription factor STAT6. J Allergy Clin Immunol. (2023) 152(1):182–94.e7. 10.1016/j.jaci.2023.01.023 36758835 PMC10330134

[74] Kallies A Good-Jacobson KL. Transcription factor T-bet orchestrates lineage development and function in the immune system. Trends Immunol. (2017) 38(4):287–97. 10.1016/j.it.2017.02.003 28279590

[75] Bennett CL Christie J Ramsdell F Brunkow ME Ferguson PJ Whitesell L The immune dysregulation, polyendocrinopathy, enteropathy, X-linked syndrome (IPEX) is caused by mutations of FOXP3. Nat Genet. (2001) 27(1):20–1. 10.1038/83713 11137993

[76] Ben-Skowronek I. IPEX syndrome: genetics and treatment options. Genes. (2021) 12(3):323. 10.3390/genes12030323 33668198 PMC7995986

[77] Barzaghi F Passerini L Bacchetta R. Immune dysregulation, polyendocrinopathy, enteropathy, x-linked syndrome: a paradigm of immunodeficiency with autoimmunity. Front Immunol. (2012) 3:211. 10.3389/fimmu.2012.00211 23060872 PMC3459184

[78] Lin W Haribhai D Relland LM Truong N Carlson MR Williams CB Regulatory T cell development in the absence of functional Foxp3. Nat Immunol. (2007) 8(4):359–68. 10.1038/ni1445 17273171

[79] Charbonnier LM Cui Y Stephen-Victor E Harb H Lopez D Bleesing JJ Functional reprogramming of regulatory T cells in the absence of Foxp3. Nat Immunol. (2019) 20(9):1208–19. 10.1038/s41590-019-0442-x 31384057 PMC6707855

[80] Van Gool F Nguyen MLT Mumbach MR Satpathy AT Rosenthal WL Giacometti S A mutation in the transcription factor Foxp3 drives T helper 2 effector function in regulatory T cells. Immunity. (2019) 50(2):362–77.e6. 10.1016/j.immuni.2018.12.016 30709738 PMC6476426

[81] Du J Wang Q Yang S Chen S Fu Y Spath S FOXP3 exon 2 controls treg stability and autoimmunity. Sci Immunol. (2022) 7(72):eabo5407. 10.1126/sciimmunol.abo5407 35749515 PMC9333337

[82] Passerini L Barzaghi F Curto R Sartirana C Barera G Tucci F Treatment with rapamycin can restore regulatory T-cell function in IPEX patients. J Allergy Clin Immunol. (2020) 145(4):1262–71.e13. 10.1016/j.jaci.2019.11.043 31874182

[83] Baud O Goulet O Canioni D Le Deist F Radford I Rieu D Treatment of the immune dysregulation, polyendocrinopathy, enteropathy, X-linked syndrome (IPEX) by allogeneic bone marrow transplantation. N Engl J Med. (2001) 344(23):1758–62. 10.1056/NEJM200106073442304 11396442

